# One-Carbon Metabolism Biomarkers and Risks of Incident Neurocognitive Disorder among Cognitively Normal Older Adults

**DOI:** 10.3390/nu14173535

**Published:** 2022-08-27

**Authors:** Paulina Maria Przybycien-Gaweda, Tih Shih Lee, Wee Shiong Lim, Mei Sian Chong, Philip Yap, Chin Yee Cheong, Iris Rawtaer, Tau Ming Liew, Xinyi Gwee, Qi Gao, Keng Bee Yap, Tze Pin Ng

**Affiliations:** 1 Department of Psychological Medicine, Yong Loo Lin School of Medicine, National University of Singapore, Singapore 119228, Singapore; 2 Laboratory of Neurobehavioural Genomics, Duke-NUS Medical School, Singapore 169857, Singapore; 3 Institute of Geriatrics and Active Aging, Tan Tock Seng Hospital, Lee Kong Chian School of Medicine, Nanyang Technological University, Singapore 308433, Singapore; 4 Geriatric Education and Research Institute, Singapore 768024, Singapore; 5 Department of Geriatric Medicine, Khoo Teck Puat Hospital, Singapore 768828, Singapore; 6 Department of Psychiatry, Sengkang General Hospital, Singapore 544886, Singapore; 7 Department of Psychiatry, Singapore General Hospital, Singapore 169608, Singapore; 8 Department of Clinical Epidemiology, Tan Tock Seng Hospital, Singapore 308433, Singapore; 9 Department of Medicine, Ng Teng Fong General Hospital, Singapore 609606, Singapore

**Keywords:** folate, B12, homocysteine, mild cognitive impairment, dementia

## Abstract

There is a lack of evidence supporting an association between folate and vitamin B12 exposure with cognitive outcomes. We examined serum folate and vitamin B12 and plasma homocysteine in 690 cognitively-normal adults (aged ≥ 55) from the Singapore Longitudinal Aging Study (SLAS-2) followed-up over 4.5 years on incident neurocognitive disorder (NCD): mild cognitive impairment (MCI) and dementia. At follow-up, 5.7% (39) of participants developed NCD (34 MCI and 5 dementia). Comparing with those who remained cognitively-normal, participants progressed to NCD had significantly lower mean baseline vitamin B12 (420 [SD ± 221] vs. 510 [SD ± 290] pmol/L, *p* = 0.026), higher homocysteine (14.6 [SD ± 4.2] vs. 12.9 [SD ± 4.3], *p* = 0.018) and lower one-carbon index (Z-scores: −0.444 [SD ± 0.819] vs. −0.001 [SD ± 0.990], *p* = 0.006). Adjusted for confounders, significant associations with incident NCD were found for lower vitamin B12 (per-SD OR = 2.10, 95%CI = 1.26–3.52), higher homocysteine (per-SD OR = 1.96, 95%CI = 1.18–3.24) and lower one-carbon index (per-SD OR = 1.67, 95%CI = 1.06–2.64). Folate was not significantly associated with progression to NCD. Notably, low B12 in the presence of high folate was significantly associated with incident NCD (adjusted OR = 3.81, 95%CI = 1.04–13.9). Low B12, high homocysteine, low B12 in the presence of high folate, and a one-carbon index of hypo-methylation were independently associated with progression to NCD among cognitively normal.

## 1. Introduction

The study of blood biomarkers that predict future risks of neurocognitive disorders (NCD) which include mild cognitive impairment (MCI) and dementia is a dynamically growing field. The identification of blood biomarkers could facilitate early recognition of an at-risk state and targeted interventions to prevent cognitive deterioration [1].

One group of potential risk factors and blood biomarker predictors of the risk of NCD are folate, B12 and homocysteine, which are involved in one-carbon (1-C) metabolism [2]. 1-C metabolism describes a group of reactions that provide methyl groups in amino acid and homocysteine/methionine production cycles, which are essential in the synthesis of monoamine neurotransmitters in brain tissues. B-vitamins such as folate, vitamin B2, and B6 are the key co-enzymes and co-factors for these reactions [3,4].

Cross-sectional studies have reported that high vitamin B12 and folate concentrations are positively associated with cognition, but substantial evidence from prospective studies supporting this association is lacking [5,6,7,8]. There have been contradictory reports of longitudinal associations of low folate and low vitamin B12 [5,6,7,8,9,10,11,12,13,14,15,16,17,18,19] with cognitive decline or progression to dementia, although the evidence is more consistent for risk predicted by elevated homocysteine [20,21]. Interestingly, there have also been indications that the combination of low B12 with high folate has particularly deleterious effects on haematological and cognitive outcomes, through a postulated impaired activity of B12-dependent enzymes [22,23,24,25], although this has not been reliably replicated [13]. Further research is therefore needed to establish these relationships.

This study examines folate, B12 and homocysteine as potential predictors of NCD (MCI or early dementia] in a cohort of over-55-year-olds participating in the Singapore Longitudinal Aging Study (SLAS), who were followed up for a mean of 4.5 years.

## 2. Materials and Methods

Study design and population. Details of the SLAS have been described in previous publications [26,27]. The present study is based on data from the second recruitment cohort (SLAS-2) of 3270 participants who were recruited between 2010 and 2013 from the South-West and South-Central regions of Singapore. The study was approved by the National University of Singapore Institutional Review Board. All participants gave written informed consent. The participants were identified by the door-to-door census, with a response rate of 78.5%. Community-dwelling over-55-year-olds who were self-ambulatory and possessed sufficient cognitive capacity for participation were included. Individuals with severe physical or mental disabilities were excluded.

Measurements at baseline included a comprehensive clinical assessment, structured questionnaire interviews, neurocognitive and functional testing and blood sampling to collect an extensive range of socioeconomic, lifestyle, behavioral, cognitive and physiological data [26,27]. Blood samples were collected after overnight fasting, and a simple probability sampling procedure was used to randomly select two-thirds of the blood samples in SLAS2 (*N* = 1770) for analysis of serum folate, B12 and plasma total homocysteine concentrations.

In this sub-cohort with available blood biomarker data, 1507 participants were cognitively normal at baseline and 690 (46%) participants completed the follow-up study (305 participants died and 512 were uncontactable or refused further participation (see Supplementary Figure S1 in the supplement). The mean follow-up time was 4.5 years.

Measurements. Assessments of cognitive status at baseline and follow-up have been detailed in previous publications and are briefly described here. Initial screening for cognitive impairment was performed using self or informant reports of subjective cognitive decline (IQCODE) [28] and a locally validated translated version of the Mini-Mental State Examination (MMSE) with a screening threshold of 26/27 [29]. Screened participants were further assessed using the Clinical Dementia Rating (CDR) Scale [30] and neuropsychological testing of five cognitive domains: memory, executive function, language, visuospatial skills and attention, using a battery of neuropsychological tests [31]. The final clinical assessments included magnetic resonance imaging (MRI) and consensus diagnosis by a panel of geriatricians and psychiatrists.

MCI was defined according to published criteria [32]: cognitive concern expressed by the participant or informant, impairment in ≥1 cognitive domains, normal functional ability and absence of dementia. This was operationally determined by (1) subjective memory and cognitive difficulties, or the Informant Questionnaire on Cognitive Decline in the Elderly (IQCODE) score > 3.3; (2) objective cognitive impairment in 1 or more domains: MMSE global score ranging from 24 to 27, or a decline of MMSE ≥ 2 points from baseline; and at least one neurocognitive domain (attention, memory, executive function, language or visuospatial abilities) score that were 1 to 2 standard deviations (SDs) less than the age and education-adjusted mean values, or drop from baseline of 0.5 SD during follow-up assessments; (3) CDR global score ≥ 0.5; (4) essentially independent in performing basic Activities of Daily Living (BADL); and (5) not demented.

Dementia was diagnosed based on the Diagnostic and Statistical Manual of Mental Disorders [33], with evidence of cognitive deficit (MMSE ≤ 23 or neuropsychological domain scores < 2 SD of age-education-adjusted mean), and evidence of functional impairment (dependency in ≥ 1 activities of daily living or CDR score ≥ 1).

Cognitive outcomes. Participants who had either MCI or dementia were classified as having NCD. Participants who did not meet the criteria for MCI or dementia were classified as cognitively normal.

Blood biomarkers. Venous blood samples were drawn between 0900 and 0930 after fasting overnight according to standard procedures. Blood samples were transported to Singapore National University Hospital Referral Laboratory on ice within 2 h and stored at −80 °C before analysis. Folate and vitamin B12 were measured in serum using a radioimmunoassay with Elecsys Folate II reagent kit (coefficient of variation (CV) range 6.1–13.8%) and Elecsys Vitamin B12 reagent kit (CV range 3.2–7.6%), (Roche Diagnostic, Indianapolis, IN). Plasma total homocysteine (tHcy) was measured using an automated chemiluminescent enzyme immunoassay method (CV range 4.1–10.4%) (Diagnostic Products Corporation, Los Angeles, CA, USA). APOE genotyping was identified by polymerase chain reaction (PCR) amplification followed by restriction endonuclease digestion of the PCR product (PCR-restriction fragment length polymorphism) [34].

Baseline co-variables included known psychosocial, lifestyle and health risk factors for neurocognitive disorder: age, sex, ethnicity (Chinese versus non-Chinese), education (6 years of schooling); physical, social and productive activity scores based on the number and frequencies on a 3-point Likert scale of usual participation in 18 different categories of activities; apolipoprotein E-ε4 allele, Geriatric Depression Scale (GDS) score of ≥5 [29]. Cardio-metabolic risk factors included central obesity (waist circumference ≥90 cm for males and ≥80 cm for females); high fasting blood glucose or diabetes (FBG ≥ 5.6 mmol/L, or on treatment for type 2 diabetes), hypertension (systolic blood pressure ≥ 130 or diastolic blood pressure ≥85 mmHg, or on treatment for hypertension), low high-density lipoprotein cholesterol (HDL-C) level (<1.0 mmol/L); high triglycerides (>2.2 mmol/L) and MetS was diagnosed by the presence of three or more criteria of the National Cholesterol Education Program Adult Treatment Panel (NCEP-ATP) III criteria. Serum cholesterol and triglycerides were measured using standard enzymatic procedures. Plasma glucose was measured using the glucose oxidase method. History of cardiovascular diseases included heart disease or stroke.

Statistical analysis. Continuous measures of the levels of folate, vitamin B12 and homocysteine were log10-transformed and divided into quartiles based on approximate SD intervals. The latter allows the estimated strengths of association with progression to NCD to be directly compared based on a standardized per-SD score. Clinically adopted measures of low folate, B12 and high homocysteine based on published cut-offs or laboratory reference values were used to divide the participants into low folate, low B12, high homocysteine groups, and three mutually exclusive and collectively exhaustive B12-folate subgroups: low B12 and high folate, low B12 or low folate, and normal B12 and normal folate. Given the dynamic inter-relationship among folate, B12 and homocysteine, we also used principal component analysis (PCA) to create a one-carbon index combining the three biomarkers, with four quartile categories of z-values (≤−1.00, −0.99–0.00, 0.01–1.00 and ≥1.01) and low values indicating hypo-methylation.

Differences in baseline characteristics between participants who exhibited cognitive decline or progressed to NCD at follow-up and those who did not were compared using ANOVA tests for continuous variables and chi-squared tests for categorical variables. Binary logistic regression was used to estimate the strength of associations of blood biomarkers with incident NCD. Adjusted odds ratios (ORs) were estimated using a model adjusted for the baseline co-variables, as described above. ORs were estimated with their 95% confidence intervals (CIs). Data analysis was performed using IBM-SPSS version 25. All statistical significance tests were two-sided, and an α-level of 0.05 was considered significant.

## 3. Results

Among the 690 cognitively normal participants who had available baseline blood test results, the mean age was 64.9 years (SD ± 6.6); 5.1% (*N* = 35) participants were non-Chinese and the remaining participants were Chinese; 64.1% (*N* = 442) were women. The demographic and risk factor profile of study participants are shown in Table 1. At follow-up, 5.7% (*N* = 39) progressed to NCD (34 progressed to MCI and 5 progressed to dementia).

Baseline risk factor profile. The baseline profile of sociodemographic and medical risk factors of participants according to their cognitive status at follow-up is shown in Table 1. Participants who progressed to NCD at follow-up were significantly more likely to be older, non-Chinese, female, less educated, have a lower physical and overall activity score and have metabolic syndrome.

**Figure 1:**
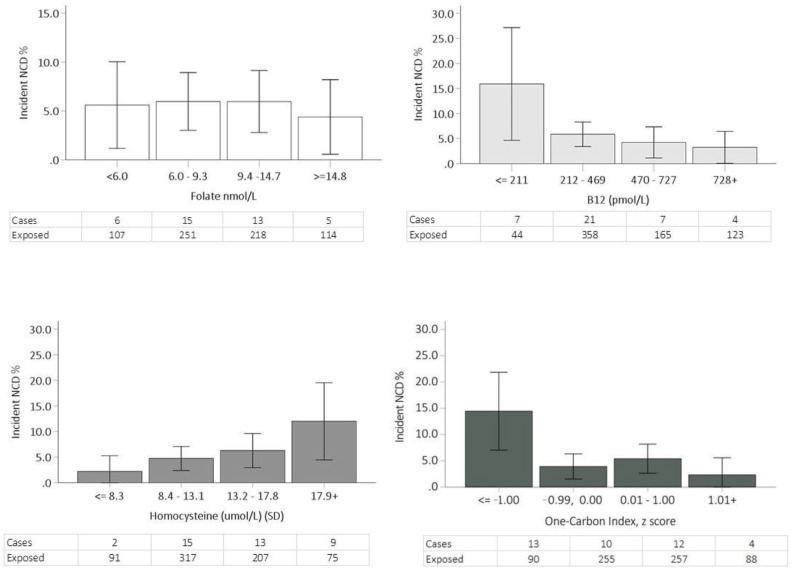
Number and % of incident neurocognitive disorder by SD quartiles of folate, B12, homocysteine and one-carbon index.

Blood biomarkers and NCD. In comparison to the participants who remained cognitively normal at follow up, participants who progressed to NCD had significantly lower mean baseline vitamin B12 (420 [SD ± 221] vs. 510 [SD ± 290] pmol/L, *p* = 0.026) and one-carbon index (Z-scores: −0.444 [SD ± 0.819] vs. −0.001 [SD ± 0.990], *p* = 0.006) but higher homocysteine (14.6 [SD ± 4.2] vs. 12.9 [SD ± 4.3], *p* = 0.018) (Table 1, Figure 1). They were also more likely to be in the two lowest quartiles for vitamin B12 level (17.9% [*N* = 7] vs. 5.7% [*N* = 37] for ≤211 pmol/L and 53.8% [*N* = 21] vs. 51.8% [*N* = 337] for 212–469 pmol/L; *p* = 0.014), in the lowest quartile for one-carbon index (33.3% [*N* = 13] vs. 11.8% [*N* = 77] for Z-score ≤ −1.00; *p* = 0.007) and in the two highest quartiles for homocysteine (23.1% [*N* = 9] vs. 10.1% [*N* = 66] for ≥17.9 μmol/L and 33.3% [*N* = 13] vs. 29.8% [*N* = 194] for 13.2–17.8 μmol/L; *p* = 0.007). Folate level at baseline was not associated with progression to NCD at follow-up.

Among the participants who progressed to NCD there was also a higher percentage of those with low vitamin B12-and-high folate (10.3% [*N* = 4] vs. 2.8% [*N* = 18]) and with either low B12 or low folate (38.5% [*N* = 15] vs. 30.9% [*N* = 201]), while the percentage of participants with normal B12 and normal folate was lower in the NCD progression group (51.3% [*N* = 20] vs. 66.4% [*N* = 432]) (*p* = 0.015).

Logistic regression analysis. We estimated ORs of association of blood biomarkers with progression to NCD (Table 2) in logistic regression analysis. Lower per-SD levels of B12, one-carbon index and higher homocysteine were significantly associated with progression to NCD in crude analyses. After adjustment for confounding co-variables, the association with NCD for lower levels of vitamin B12 (per-SD OR = 2.10, 95%CI = 1.26–3.52), for higher levels of homocysteine (per-SD OR = 1.96, 95%CI = 1.18–3.24), and for lower levels of one-carbon index (per-SD OR = 1.67, 95%CI = 1.06–2.64) remained significant. There was no significant association with progression to NCD for folate level. Low B12-high folate (versus normal folate-and-normal B12) was significantly associated with progression to NCD in the unadjusted model (OR = 4.80 (95%CI = 1.49–15.5), and after adjustment for confounding co-variables (OR = 3.81 (95%CI = 1.04–13.9).

## 4. Discussion

In the most recent meta-analysis of studies conducted prior to 2020 of community-dwelling adults aged 45 years and above [7], overall positive associations of B12 and folate with cognitive function were firmly demonstrated in cross-sectional studies, but substantial evidence from prospective studies was lacking. Folate and vitamin B12 were found to be associated with cognitive decline in some longitudinal studies [9,10,11], but not all [12,13,14,15,16,17,18,19]. Our prospective cohort study showed that cognitively normal adults aged 55 and over who had low vitamin B12 at baseline had higher odds of progression to NCD. We also found an association between increased levels of homocysteine with higher odds of progression to NCD. This is also in agreement with most of the earlier studies [9,16,20,21].

We found no apparent association between folate and NCD risk in this study when B12 status was not taken into account. The level of folate was found to be inconsistently associated with cognition in many studies including our own. Indeed, high folate was reported in some studies to be associated with cognitive impairment or decline when the level of B12 was unknown or low [19]. On the other hand, in the presence of normal vitamin B12, studies reported associations between low folate status and impaired cognition (and vice versa). Across different studies, the failure to take into account B12 vis-à-vis folate status in the analyses, and the prevalence of relative folate and B12 deficiency and food fortification of folate in the study population may thus explain discrepant findings. Other sources of heterogeneity of findings across studies include variations in sample size, participants’ age, cognitive testing and duration of follow-up.

Levels of folate, B12 and homocysteine are intimately related to one another given their roles as cofactors in the catalytic transfer of methyl groups between homocysteine and methionine. The latter is essential for the formation of S-adenosylmethionine (SAM), a universal methyl donor in methylation reactions in the production of monoamine neurotransmitters in the brain. B12 sufficiency is required for a critical irreversible step in the transfer of the methyl group catalyzed by methionine synthesis; hence when B12 is lacking, folate accumulates [4]. Higher folate status in vitamin B12 deficiency has been found to be associated with higher circulating levels of total homocysteine, as well as anaemia and cognition. This was also observed with secondary data analysis in the present study, shown in the Supplementary Table S1 in the Supplement. Congruently, we found that low B12-high folate status (versus normal folate-and-normal B12) was significantly associated with about 4 times increased odds of progression to NCD. Using an alternative PCA approach to derive a combined measure of folate, B12 and homocysteine, we also found that lower levels of this one-carbon index indicating hypo-methylation were associated with increased odds of incident NCD, robust to adjustment for confounding variables.

While our study adds to the limited evidence from prospective studies that low vitamin B12 and high homocysteine are associated with increased risks of neurocognitive disorders, there are pertinent findings from interventional studies that should also be considered. In a recent 2018 Cochrane systematic review of 14 randomized controlled trials (RCTs) involving B-vitamin supplementation (folic acid, vitamin B12, vitamin B6 or their combinations in comparison to placebo) in cognitively normal middle-aged participants, no clinically significant effect on global cognitive function after 5 or 10 years were found [35]. It has also been shown that the level of homocysteine can be modified through B-vitamin supplementation but a lowering of homocysteine did not have the full expected clinical results such as reduction in cardiovascular events [36]. Similarly, in a 2016 meta-analysis of RCTs aiming at improving cognition in patients with diagnosed dementia, homocysteine reduction by B-vitamin supplementation resulted in no improvement in cognitive function, as measured by MMSE despite a significant fall in homocysteine levels [37]. On the other hand, in a study of over-70-year-olds with MCI, B-vitamin supplementation was found to be associated with both lower homocysteine and a slower rate of cognitive decline [38]. The interactions between B-vitamin and homocysteine and their roles in cognition are complex. Many factors may modify cognitive outcomes of B-vitamins and homocysteine-lowering exposure, including age and clinical characteristics including folate status of the target population. Considering the fact that many countries mandate folate fortification of food products, investigators and clinicians should therefore be particularly concerned about the potentially deleterious cognitive effect of high folate in the presence of low B12. Further studies, however, are necessary. The results of our study suggest B vitamins and homocysteine are potential risk factors that should remain promising targets for further interventional studies.

Strengths and limitations. As most previous longitudinal studies have used cognitive decline as the outcome measure, a strength of this study is the singular use of a consensus diagnosis of neurocognitive disorder (mild cognitive impairment and early dementia). The study population of over-55-year-olds straddles middle and old age, and most of the incident NCD cases were MCI cases (87% [*N* = 34]), a reversible pre-dementia state that is amenable to preventative interventions [39,40]. Due to the relatively young age of this cohort, the number of incident dementia cases was very few (*N* = 5), hence they were combined with MCI under the NCD label. Given the wide follow-up interval between 3 to 5 years, these cases likely progressed to dementia without an intervening MCI state being detected. The subtyping of NCD according to the underlying brain disease pathology was beyond the scope of this study. Cross-sectional and longitudinal studies have shown that blood concentrations of B12 and total homocysteine in particular are associated with increased or accelerated brain tissue volume loss and total white matter hyperintensities [38,41,42,43,44,45,46]. In addition, only a single measurement of the 1-C metabolism biomarkers was taken at baseline, whereas information on their trajectories over time could shed more light on the pathogenesis and course of cognitive outcomes. Possible selection bias at baseline and loss to follow-up should be acknowledged, as the individuals who were excluded due to death and loss to follow-up were older, less educated, lower in MMSE, and generally had more adverse lifestyle behavioral and health profiles than those who were in the follow-up study. This may have likely resulted in an under-estimation of true effects.

## 5. Conclusions

In this prospective study, low B12, but not low folate, and high homocysteine were found to be associated with increased risk of incident NCD among cognitively normal middle-aged and older adults. High folate-low B12 status and a PCA index of hypo-methylation were also found to be associated risk markers.

## Figures and Tables

**Table 1:** Baseline characteristics of cognitively normal participants by cognitive outcomes at follow up.

Characteristics		Full Sample		Remained Cognitively Normal		Progressed to NCD		*p*
		*N* = 690		*N* = 651		*N* = 39 (5) ¥		
Age		64.9	±6.6	64.7	±6.4	68.7	±9.0	<0.001 ***
Ethnicity	Non-Chinese	5.1	(35)	4.5	(29)	15.4	(6)	0.003 **
Sex	Female	64.1	(442)	63.1	(411)	79.5	(31)	0.036 *
Education	≤6 years	55.4	(382)	53.9	(351)	79.5	(31)	0.002 **
Smoking	Never	80.6	(556)	80.6	(525)	79.5	(31)	0.260
	Ex-smoker	9.3	(64)	8.9	(58)	15.4	(6)	
	Current smoker	10.1	(70)	10.4	(68)	5.1	(2)	
Alcohol	≥1 drinks daily	0.1	(1)	0.2	91)	0.0	(0)	0.807
APOE-ε4		15.7	(108)	15.2	(99)	23.1	(9)	0.189
Physical activity score	0–8	2.5	±1.6	2.6	±1.6	1.9	±1.2	0.010 *
Social activity score	0–14	3.5	±2.9	3.6	±3.0	2.7	±2.0	0.083
Productive activity score	0–9	4.3	±1.8	4.3	±1.8	4.3	±1.8	0.963
Overall activity score	0–30	10.2	±4.5	10.3	±4.6	8.7	±3.4	0.043 *
GDS score		0.59	±1.07	0.60	±1.09	0.44	±0.64	0.345
Central obesity		53.9	(372)	53.1	(346)	66.7	(26)	0.096
Hyperglycaemia/diabetes		25.9	(179)	25.3	(165)	35.9	(14)	0.104
Hypertension		62.5	(431)	62.1	(404)	69.2	(27)	0.369
Low HDL-C	<1.0 mmol/L	49.4	(341)	49.2	(320)	53.8	(21)	0.569
High TG	>2.2 mmol/L	47.0	(324)	47.3	(308)	41.0	(16)	0.445
Metabolic syndrome	≥3 components	34.6	(239)	33.8	(220)	48.7	(19)	0.044 *
Heart disease/stroke	Yes vs. No	8.8	(61)	8.6	(56)	12.8	(5)	0.367
Folate	nmol/L	10.4	±5.7	10.5	±5.8	9.7	±4.0	0.393
	<6.0	15.5	(107)	15.5	(101)	15.4	(6)	0.706
	6.0–9.3	36.4	(251)	36.3	(236)	38.4	(15)	
	9.4–14.7	31.6	(218)	31.5	(205)	33.3	(13)	
	≥14.8	16.5	(114)	16.7	(109)	12.8	(5)	
B12	pmol/L	505	±221	510	±290	420	±221	0.026 *
	≤211	6.4	(44)	5.7	(37)	17.9	7	0.014 *
	212–469	51.9	(358)	51.8	(337)	53.8	21	
	470–727	23.9	(165)	24.3	(158)	17.9	7	
	≥728	17.8	(123)	18.3	(119)	10.3	4	
Homocysteine	μmol/L	13.0	±4.3	12.9	±4.3	14.6	±4.2	0.018 *
	≤8.3	13.2	(91)	13.7	(89)	5.1	(2)	0.007 **
	8.4–13.1	45.9	(317)	46.4	(302)	38.5	(15)	
	13.2–17.8	30.0	(207)	29.8	(194)	33.3	(13)	
	≥17.9	10.9	(75)	10.1	(66)	23.1	(9)	
1-C index	Z-scores	−0.026	±0.986	−0.001	±0.990	−0.444	±0.819	0.006 **
	≤−1.00	13.0	(90)	11.8	(77)	33.3	(13)	0.007 **
	−0.99–0.00	37.0	(255)	37.6	(245)	25.6	(10)	
	0.01–1.00	37.2	(257)	37.6	(245)	35.9	(12)	
	≥ 1.01	12.8	(88)	12.9	(84)	10.3	(4)	
B12-folate subgroups	Low B12 and high folate	3.2	(22)	2.8	(18)	10.3	(4)	0.015 *
	Low B12, or low folate	31.3	(216)	30.9	(201)	38.5	(15)	
	Normal B12, normal folate	65.5	(452)	66.4	(432)	51.3	(20)	

Figures shown are mean ± standard deviation or % (*N*); ¥ Number of dementia cases in parentheses; *p*-values are from log10-transformed values of folate, B12 and homocysteine. Cut-offs are based on approximate SD intervals. * *p* < 0.05; ** *p* < 0.01; *** *p* < 0.001 by ANOVA test (for continuous variables), or chi-squared test (for categorical variables). APOE: apolipoprotein E, B12: vitamin B12, GDS: Geriatric Depression Scale, HDL-C: high-density lipoprotein cholesterol, NCD: neurocognitive disorder (mild cognitive impairment or dementia), SD: standard deviation, TG: triglycerides.

**Table 2:** Associations of B12, folate and homocysteine with incident NCD.

		Unadjusted			Adjusted †			
Predictor Variable		OR	95%CI	*p*	OR	95%CI		*p*
Folate	Per SD (reversed)	1.12	(0.58, 2.16)	0.741	0.94	(0.64, 1.39)		0.776
B12	Per SD (reversed)	1.70	(1.11, 2.61)	0.016 *	2.10	(1.26, 3.52)		0.005 **
Homocysteine	Per SD	1.68	(1.15, 2.44)	0.007 **	1.96	(1.18, 3.24)		0.009 **
1-Carbon index	Per SD (reversed)	1.56	(1.07, 2.29)	0.021 **	1.67	(1.06, 2.64)		0.027 **
Folate	<6.0	1.29	(0.38, 4.37)	0.677	0.93	(0.25, 3.45)		0.914
	6.0–9.3	1.39	(0.49, 3.91)	0.538	0.96	(0.31, 2.94)		0.947
	9.4–14.7	1.38	(0.49, 3.91)	0.548	1.16	(0.37, 3.60)		0.794
	≥14.8 nmol/L	1			1			
B12	≤211	5.63	(1.56, 20.3)	0.008 **	16.3	(2.86, 93.5)		0.002 **
	212–469	1.85	(0.62, 5.51)	0.267	3.72	(0.80, 17.2)		0.093
	470–727	1.32	(0.38, 4.61)	0.665	3.41	(0.64, 18.1)		0.149
	>727 pmol/L	1			1			
Homocysteine	<8.4	1			1			
	8.4–13.1	2.21	(0.50, 9.85)	0.298	1.17	(0.25, 5.61)		0.840
	13.2–17.8	2.98	(0.66, 13.5)	0.156	2.52	(0.51, 12.6)		0.256
	≥17.9 μmol/L	6.07	(1.27, 29.0)	0.024 *	5.27	(0.87, 31.7)		0.070
1-C Index (z-score)	≤−1.00	3.54	(1.11, 11.3)	0.033 *	6.23	1.43	27.1	0.015 **
	−0.99–0.00	0.86	(0.26, 2.80)	0.799	1.12	0.28	4.51	0.873
	0.01–1.00	1.03	(0.32, 3.28)	0.962	1.57	0.39	6.23	0.524
	≥1.01	1			1			
B12-folate	Low B12 and high folate	4.80	(1.49, 15.5)	0.009 **	3.81	(1.04, 13.9)		0.044 *
Subgroups	Low B12 and/or low or normal folate	1.61	(0.81, 3.21)	0.175	1.49	(0.73, 3.07)		0.277
	Normal B12, Normal folate	1			1			

† Adjusted for age, sex, ethnicity, education (≤6 years of schooling), smoking, physical activity, social activity, productive activity, apolipoprotein E-ε4 allele, Geriatric Depression Scale score, central obesity, high fasting blood glucose or diabetes, hypertension, low high-density lipoprotein cholesterol level, high triglycerides, heart disease or stroke; * *p* < 0.05; ** *p* < 0.01 by ANOVA test (for continuous variables), or chi-squared test (for categorical variables) B12: vitamin B12, NCD: neurocognitive disorder (mild cognitive impairment or dementia), SD: standard deviation.

## Data Availability

The datasets used and/or analysed during the current study are available from the corresponding author on reasonable request.

## Supplementary Materials

The following supporting information can be downloaded at: https://www.mdpi.com/article/10.3390/nu14173535/s1, Figure S1: Participant recruitment flowchart; Table S1: Mean ± SD levels of homocysteine, red cell parameters, MMSE global cognition, and depressive symptoms by B12-Folate subgroups.
