# Supplementary material for: One-Carbon Metabolism Biomarkers and Risks of Incident Neurocognitive Disorder among Cognitively Normal Older Adults
Source: Nutrients. 2022 Aug 27;14(17):3535. doi: 10.3390/nu14173535 (PMC9459953; doi:10.3390/nu14173535)
Supplement: Supplementary file 1 [file nutrients-14-03535-s001.zip › nutrients-1872614-supplementary.pdf]

ONLINE SUPPLEMENTARY MATERIAL

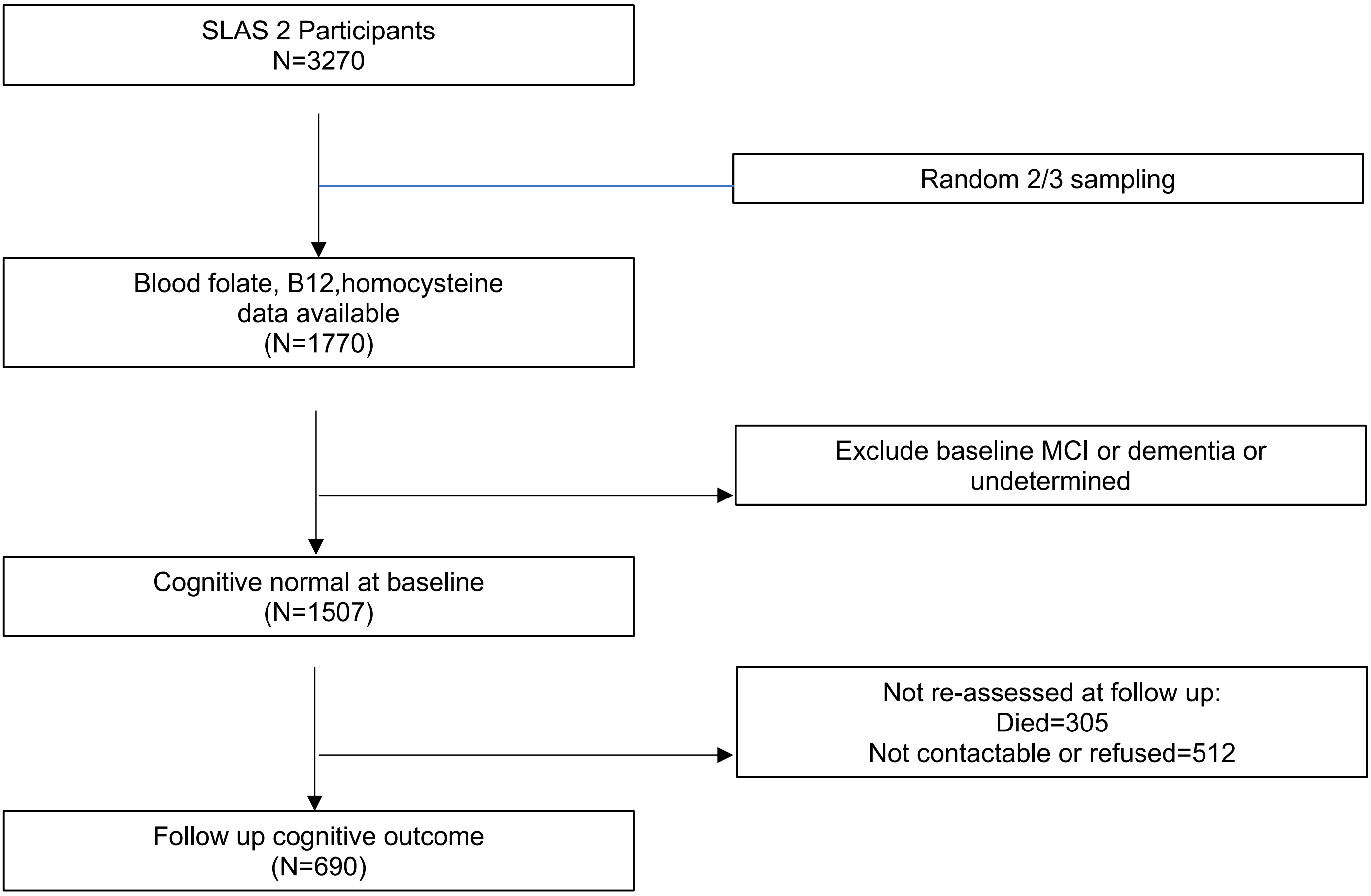

Supplementary Figure S1. Participant recruitment flowchart

Supplementary Table S1. Mean ± SD levels of homocysteine, red cell parameters, MMSE global cognition, and depressive symptoms by B12-Folate subgroups

		Normal B12, Normal folate N=452		Low B12 or Low Folate N=216		Low B12, High Folate N=22		p
Homocysteine, Hcy	umol/L	12.0	± 3.5	14.7	± 4.7	16.2	± 7.7	<0.001
Haemoglobin, Hb	g/dL	13.3	± 1.3	13.3	± 1.5	12.9	± 1.4	0.302
Red cell count, RBC	$x10^{12}/L$	4.57	± 0.51	4.60	± 0.51	4.39	± 0.46	0.175
Red cell distribution width, RDW	%	13.2	± 0.85	13.3	± 0.89	12.9	± 0.54	0.046
Mean corpuscular volume, MCV	fL	87.2	± 6.4	87.4	± 7.0	87.6	± 3.9	0.947
Mean corpuscular haemoglobin, MCH	pg	29.2	± 2.5	29.1	± 2.7	29.3	± 1.7	0.863
Mean corpuscular haemoglobin concentration	g/dL	33.5	± 1.1	33.3	± 1.2	33.5	± 1.2	0.213
Mini Mental State Examination, MMSE	0-30	29.0	± 1.2	28.7	± 1.5	27.8	± 2.3	<0.001
Geriatric depression scale, GDS	0-15	0.56	± 1.02	0.55	± 0.85	1.59	± 2.65	<0.001
